# GWLS: A Novel Model for Predicting Cognitive Function Scores in Patients With End-Stage Renal Disease

**DOI:** 10.3389/fnagi.2022.834331

**Published:** 2022-02-03

**Authors:** Yutao Zhang, Zhengtao Xi, Jiahui Zheng, Haifeng Shi, Zhuqing Jiao

**Affiliations:** ^1^School of Microelectronics and Control Engineering, Changzhou University, Changzhou, China; ^2^Department of Radiology, Changzhou Second People’s Hospital Affiliated to Nanjing Medical University, Changzhou, China; ^3^School of Computer Science and Artificial Intelligence, Changzhou University, Changzhou, China

**Keywords:** end-stage renal disease, cognitive function scores, model, functional magnetic resonance imaging, predict

## Abstract

The scores of the cognitive function of patients with end-stage renal disease (ESRD) are highly subjective, which tend to affect the results of clinical diagnosis. To overcome this issue, we proposed a novel model to explore the relationship between functional magnetic resonance imaging (fMRI) data and clinical scores, thereby predicting cognitive function scores of patients with ESRD. The model incorporated three parts, namely, graph theoretic algorithm (GTA), whale optimization algorithm (WOA), and least squares support vector regression machine (LSSVRM). It was called GTA-WOA-LSSVRM or GWLS for short. GTA was adopted to calculate the area under the curve (AUC) of topological parameters, which were extracted as the features from the functional networks of the brain. Then, the statistical method and *Pearson* correlation analysis were used to select the features. Finally, the LSSVRM was built according to the selected features to predict the cognitive function scores of patients with ESRD. Besides, WOA was introduced to optimize the parameters in the LSSVRM kernel function to improve the prediction accuracy. The results validated that the prediction accuracy obtained by GTA-WOA-LSSVRM was higher than several comparable models, such as GTA-SVRM, GTA-LSSVRM, and GTA-WOA-SVRM. In particular, the root mean square error (RMSE), mean absolute error (MAE), and mean absolute percentage error (MAPE) between the predicted scores and the actual scores of patients with ESRD were 0.92, 0.88, and 4.14%, respectively. The proposed method can more accurately predict the cognitive function scores of ESRD patients and thus helps to understand the pathophysiological mechanism of cognitive dysfunction associated with ESRD.

## Introduction

End-stage renal disease (ESRD) refers to the most severe stage of chronic kidney disease. At this stage, the glomerular filtration rate of the patient is less than 15 ml/min⋅(1.73 m^2^), and the patient needs long-term dialysis or kidney transplantation to maintain life (Drew et al., 2017; Balbino et al., 2021). Studies have shown that patients with ESRD generally have accompanying symptoms of cognitive dysfunction, such as thinking retardation, insensitivity, inattention, and memory loss. Simultaneously, patients are also accompanied by severe negative emotions such as anxiety and depression (Emma et al., 2016; Zhao et al., 2019). The internationally recognized “kidney-brain” axis theory may explain these accompanying symptoms (Miranda et al., 2017). The brain and kidney have similar hemodynamics; therefore, the patients with ESRD are prone to small vessel injury of the brain. The long-term accumulation of uremia toxin causes the disorder of brain metabolism in patients with ESRD, and these factors may cause the cognitive dysfunction in patients with ESRD (Bugnicourt et al., 2013). The study of the structural and functional impairment of the brain in patients with ESRD may help understand the pathophysiological mechanism of cognitive dysfunction associated with ESRD (Li et al., 2021c).

At present, neuroimaging technology is developing rapidly. Diffusion tensor imaging (DTI), diffusion Kurtosis imaging (DKI), magnetic resonance imaging (MRI), functional magnetic resonance imaging (fMRI), electroencephalogram (EEG), and magnetoencephalography (MEG) are widely used in the diagnosis of cognitive function in patients with ESRD (Gregory and Scahill, 2018; Raurale et al., 2021). FMRI, EEG, and MEG images are used to capture the functional networks of the brain of patients with ESRD, to explore the potential relationship between the cognitive dysfunction of patients with ESRD patients and the changes in the central nervous structure of the brain (Wang et al., 2021). FMRI uses MRI to measure changes in hemodynamics caused by the neuronal activity and can detect dynamic changes in the brain in real time (Jiao et al., 2021a). Compared with EEG and MEG, fMRI has a higher spatial and temporal resolution. fMRI can be used to construct the functional network of the brain of patients with ESRD, which can more effectively help doctors or researchers understand the subtle changes in the brain of patients with ESRD.

The Montreal cognitive assessment (MoCA) can be used for rapid screening of cognitive abnormalities in patients with ESRD, and its scores can effectively help doctors to evaluate and predict the cognitive function of patients (Jiang et al., 2021). However, the educational level and emotional state of patients, skills and experience of examiners in using MoCA, and the examination environment all affect the cognitive function scores of patients (Potocnik et al., 2020). Therefore, an accurate prediction of scores of cognitive function plays an important role in subsequent treatment of patients. Wu et al. (2020) used statistical methods to analyze the correlation between the topological attribute parameters of the functional network of the brain in patients with ESRD and the score of the cognitive function. They mainly focus on the biological markers that affect the cognitive function of patients with ESRD and cannot predict the current state of the cognitive function of patients well. Yang et al. (2019) proposed a model to explore the relationship between the MRI data and the score of the cognitive function, using the longitudinal MRI data to predict the scores of the cognitive function at future time points, using the scores to determine the current cognitive function of the patients. However, the MRI generates static images for whole-body research, and it cannot show the dynamic changes of the brain activity. Lu et al. (2017) proposed a method for predicting the value of clinical variables based on the functional network of the brain, using support vector regression machines (SVRMs) to predict the scores of the cognitive function, but SVRM has the problems of certain volatility and low accuracy in the process of predicting the scores of the cognitive function.

As discussed earlier, we proposed to build a novel model for predicting the scores of the cognitive function of patients with ESRD. It is committed to exploring the relationship between the fMRI data and clinical scores of patients with ESRD. The main work is as follows. First, the graph theoretic algorithm (GTA) was adopted to calculate the area under the curve (AUC) of global topological parameters, which were extracted as the features from the functional networks of the brain. Then, the statistical method and *Pearson* correlation analysis were used to select the features. Finally, the least squares support vector regression machine (LSSVRM) was built according to the selected features to predict the scores of the cognitive function of patients with ESRD. Meanwhile, the whale optimization algorithm (WOA) was introduced to optimize the parameters in the LSSVRM kernel function to improve the prediction accuracy. The model called GTA-WOA-LSSVRM, or GWLS for short, was expected to predict the scores of the cognitive function of patients with ESRD more accurately and then find biological markers on judging their current state of the cognitive function.

## Data and Methods

### Research Framework

**FIGURE 1:**
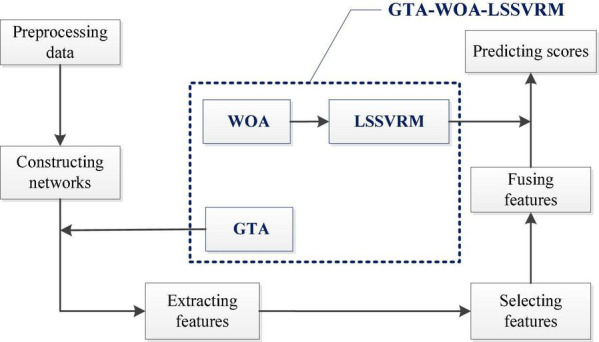
Research framework.

Figure 1 shows our research framework, which mainly includes the following steps. (A) Preprocessing the original resting-state fMRI data (Xu et al., 2019); (B) constructing the functional networks of the brain according to the time series, which were extracted from the preprocessed data; (C) adopting GTA to extract the AUC of the topological attribute parameters of the functional networks of the brain of patients with ESRD as features; (D) comparing the differences in the features between the patients with ESRD and normal controls through statistical methods; (E) calculating the *Pearson* correlation coefficient between the features and the cognitive function scores of patients with ESRD; (F) selecting features, which were significantly different from normal controls and correlated with cognitive function scores highly; (G) fusing the selected features to build LSSVRM; (H) introducing WOA to optimize the selection strategy of kernel function parameters in LSSVRM; (I) predicting the cognitive function scores of patients with ESRD through GTA-WOA-LSSVRM.

### Experimental Data and Pretreatment

A total of 45 patients with ESRD were admitted to Changzhou Second People’s Hospital Affiliated to Nanjing Medical University from February 2021 to September 2021, including 25 male and 20 female individuals, aged 49.24 ± 8.57 years. Synchronously, a total of 30 normal controls were also admitted to the same hospital, including 15 male and 15 female individuals, aged 48.20 ± 6.91 years. There were no significant differences (*p*> 0.05) in the gender ratio, age, and education level between them. One hour before fMRI examination, cognitive functions of all subjects were assessed by trained neurologists who did not know the data of subjects through MoCA. Table 1 gives the demographic information of these two groups of subjects.

**TABLE 1:** Demographic information of subjects.

	Gender (male/female)	Age (years, $\bar{x}\pm s$)	Education years (years, $\bar{x}\pm s$)	MoCA scores (points, $\bar{x}\pm s$)
ESRD patients (*n* = 45)	25/20	49.24 ± 8.57	11.47 ± 2.09	21.33 ± 2.44
Normal controls (*n* = 30)	15/15	48.20 ± 6.91	11.36 ± 2.01	27.37 ± 1.33
*t/*χ^2^	0.302	1.090	0.382	−13.809
*P*	0.583	0.279	0.731	0.000

All subjects underwent scan using a Philips 3.0T scanner and were asked to relax as much as possible to keep their brain in a resting state. Each subject was scanned for 7 min, the large frame displacement (FD > 0.5) was greater than 2.5 min, the repetition time (TR) was 3,000 ms, the number of fMRI layers was 6,720, and the layer thickness was 3.3 mm. The data-processing assistant for resting-state fMRI (DPARSF), available at http://rfmri.org/dpabi, was used to preprocess the collected original fMRI data of two types of subjects. The specific steps are as follows: (A) Converting the image format; (B) removing the first 10 time points (it takes a certain amount of time for the instrument to be checked and the subject to enter the stable state); (C) conducting the time and head movement correction; (D) conducting spatial standardization: An EPI template was used to register the standard brain space of Montreal Neurological Institute (MNI), and the voxels were resampled with a resolution of 3 mm × 3 mm × 3 mm; (E) smoothing Gaussian kernel *via* full-width-at-half-maximum; (F) removing linear drift; (G) carrying out bandpass filtering, the frequency range was 0.01–0.08 Hz; and (H) obtaining the final time series by removing the mean blood oxygenation level dependent (BOLD) time series of head motion parameters, white matter, and cerebrospinal fluid.

The brain of each subject was divided into 90 brain regions by automated anatomical labeling (AAL) standard partition template, and the *Pearson* correlation coefficient between the time series of two brain regions was calculated to construct a 90 × 90 symmetric matrix with all 1 s on the diagonal. Using the Fisher *Z* transformation, the Pearson correlation coefficient was converted to *Z*-value close to normal distribution, thus generating *Z* matrix. Taking matrix sparsity as the threshold, the *Z* matrix was binarized. In this study, the matrix sparsity was set to 0.1–0.4 with a span of 0.01. Within the threshold range of the matrix sparsity, GTA was adopted to calculate the topological attribute parameters of the functional networks of the brain, including global efficiency (E_global_), local efficiency (E_local_), clustering coefficient (C_p_), characteristic path length (L_p_), standardized clustering coefficient (γ), standardized characteristic path length (λ), and small-world properties (σ), in patients with ESRD and normal controls (Jiao et al., 2021b). The GRETNA software was used to automatically calculate the AUC of each topology attribute parameter within the entire matrix sparsity threshold.

### Principle of Least Squares Support Vector Regression Machine

Least squares support vector regression machine is an improvement on SVRM. The inequality constraint in the SVRM model is changed into equality constraint, and the solution of quadratic programming problem is transformed into the solution of linear equations, which improve the prediction efficiency. Meanwhile, it takes the error square and loss function as the experience loss of the training set, which improves the prediction accuracy and helps to effectively fit the scores of the cognitive function with non-linear characteristics (Liu et al., 2019). The specific steps are as follows:

Suppose a set of training samples is given (Yang et al., 2021):


(1) $$S=\left\{\left(x_{i},y_{i}\right),x_{i}\in R^{n},y_{i}\in R\right\},i=1,2,\cdots ,N$$

where *x*_*i*_ is the *i*-th input vector; *y*_*i*_ is the *i*-th output vector; *n* is the dimension of the input vector; *N* is the number of the training sample.

The core principle of LSSVRM is to map training samples to high-dimensional feature space through the non-linear mapping and then, perform the linear regression in a high-dimensional space. The regression function can be described as follows (Zheng et al., 2019):


(2) f(x)=ω⋅φ(x)+b

where ω is the weight vector; *φ*(*x*) is the kernel function of LSSVRM, and it represents the mapping between low-dimensional feature space and high-dimensional feature space; *b* is the amount of deviation.

According to the principle of minimizing the structural risk, the optimization problem of LSSVRM can be translated into (Shen et al., 2020):


(3) $$\left\{ \begin{array}{c} \min J\left(\omega ,b,e\right)=\frac{1}{2}{||\omega ||}^{2}+\frac{1}{2}\gamma \sum_{i=1}^{n}e_{i}^{2}\\ s.t.y_{i}=\omega^{T}\varphi \left(x_{i}\right)+b+e_{i} \end{array}\right.$$

where *e*_*i*_ is the fitting error; γ is the penalty factor, controlling the penalty degree of error. Lagrange multiplier λ_*i*_ is introduced to solve the above optimization problem (Yang, 2021):


(4) $$L\left(\omega ,b,e,\lambda \right)=J\left(\omega ,b,e\right)-\sum_{i=1}^{N}\lambda_{i}\left[\omega^{T}\varphi \left(x_{i}\right)+b+e_{i}-y_{i}\right]$$

Formula (4) is solved and derived according to *Karush-Kuhn-Tucker* conditions (Reng, 2013):


(5) $$\left\{ \begin{array}{c} \frac{\partial J}{\partial \omega }=0\to \sum_{i=1}^{i}\lambda_{i}\varphi \left(x_{i}\right)\\ \frac{\partial J}{\partial \omega }=0\to \sum_{i=1}^{i}\lambda_{i}=0\\ \frac{\partial J}{\partial \omega }=0\to \lambda_{i}=\gamma e_{i}\\ \frac{\partial J}{\partial \omega }=0\to \omega^{T}\varphi \left(x_{i}\right)+B+e_{i}-y_{i}=0 \end{array}\right.$$

By solving, _ω_ and *e* in the above equations are eliminated, and the predictive model function is finally obtained:


(6) $$f\left(x\right)=\sum_{i=1}^{N}\lambda_{i}K\left(x_{i},y_{i}\right)+b$$

where _*K(x_i_, y_i_)*_ is the kernel function, representing the non-linear mapping from an input space to a high-dimensional feature space.

As a common kernel function, the radial basis kernel function is radial symmetric and has a strong generalization ability. It can be used as the kernel function of the proposed predictive model, as shown in the following formula:


(7) $$K\left(x_{i},y_{i}\right)=\exp\left[\frac{-{||x-x_{k}||}^{2}}{2\sigma^{2}}\right]$$

where _σ_ is the width factor of the kernel function.

In LSSVRM, γ reflects the error size and the generalization ability of the model, and σ reflects the distribution characteristics of training data samples. These two parameters directly affect the prediction effect. Therefore, it is necessary to select the intelligent optimization algorithm to optimize these two parameters before prediction (Li et al., 2020b, 2021a).

### Principle of Whale Optimization Algorithm

The whale optimization algorithm is introduced to optimize the selection strategy of kernel function parameters and improve the operating efficiency of the LSSVRM model (Zhang et al., 2018; Liu et al., 2021). This algorithm is inspired by biology, and its basic principle comes from the feeding mechanism of the bubble net of humpback whales in the ocean. There are three steps included in WOA, namely, surround the prey, bubble net attack, and hunt the prey.

A. Surround the prey: in the whale algorithm, individual whales first conduct a random search based on their initial location. In mathematics, this search corresponds to the global exploration stage of the algorithm, and its mathematical model is shown in the following formulas:


(8) X→(t+1)=X→(t)*-M→⋅P→

(9) P→=|C→⋅X→(t)*-X→(t)|

(10) $$\vec{M}=2\vec{m}\cdot \vec{q}-\vec{m}$$

(11) $$\vec{N}=2\cdot \vec{q}$$

For the *t-*th iteration, “| |” is the absolute value computing; “⋅” is the dot product operation. The meanings of other parameters are as follows: $\vec{M}$ and $\vec{N}$ are the coefficient vectors of the algorithm; $\vec{X}{}^{*}$ is the location of individual whales selected at random; $\vec{X}$ is the current individual position of the whale. As the iteration progresses, $\vec{m}$ decreases linearly from 2 to 0. $\vec{q}$ is a random vector whose value is *rand* [0, 1]. The ${\vec{X}}^{*}$ of individual fish is updated in each iteration when a better position appears.

B. Bubble net attack: described by the spiral equation during the whale movement.


(12) $$\vec{X}\left(t+1\right)={\vec{P}}^{\prime }\cdot e^{bl}\cdot \cos\left(2\pi l\right)+{\vec{X}}^{*}\left(t\right)$$

(13) $${\vec{P}}^{\prime }=\left|\vec{X}\left(t\right)-{\vec{X}}^{*}\left(t\right)\right|$$

where *b* is a constant; *l* is a random number, and its value method is *rand*_[–1,1]_.

C. Search for prey: when the range of the parameter vector $\vec{M}$ is in _[–1,1]_, the optimization algorithm starts the forced search agent mechanism, and the search range is far away from the reference whale in the population. Then, a random individual is selected as the best agent to complete the update using its position, which is denoted as ${\vec{X}}_{rand}$. This mechanism ensures a better global searching ability of the algorithm. The mathematical expression of the above process is shown in the following formulas:


(14) $${\vec{p}}^{n}=\left|\vec{N}\cdot {\vec{X}}_{rand}-\vec{X}\right|$$

(15) $$\vec{X}\left(t+1\right)={\vec{X}}_{rand}-\vec{M}\cdot {\vec{P}}^{n}$$

**FIGURE 2:**
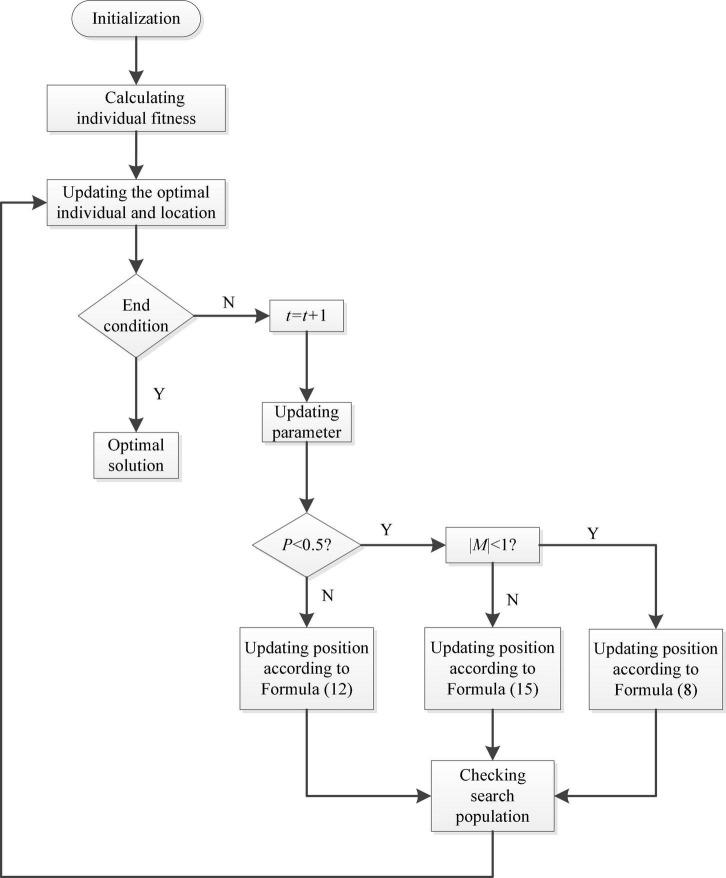
Flowchart of WOA.

Combined with the above steps, Figure 2 shows a flowchart of WOA.

## Results

### Experimental Settings

Table 2 shows the AUC of topology attribute parameters of the functional networks of the brain of patients with ESRD and normal controls calculated by GTA. Within the whole matrix sparsity threshold range, the AUC of γ and σ in patients with ESRD was significantly lower than those in normal controls, with statistical significance (*p* < 0.05). However, there were no significant differences (*p* > 0.05) in AUC of _λ_, C_p_, L_p_, E_global_, and E_local_.

**TABLE 2:** AUC of topological parameters of the functional networks of the brain between patients with ESRD and normal controls (_x̄ ± s_).

Parameter	ESRD patients (*n* = 45)	Normal controls (*n* = 30)	*t*	*P*
_γ_	0.654 ± 0.058	0.694 ± 0.032	−3.473	0.001
_λ_	0.324 ± 0.009	0.323 ± 0.008	0.289	0.773
_σ_	0.599 ± 0.053	0.635 ± 0.028	−3.384	0.001
Cp	0.174 ± 0.013	0.175 ± 0.013	0.313	0.756
Lp	0.539 ± 0.020	0.537 ± 0.017	0.456	0.650
Eglobal	0.171 ± 0.005	0.172 ± 0.004	−0.477	0.635
Elocal	0.230 ± 0.007	0.231 ± 0.006	−0.968	0.336

Table 3 shows the *Pearson* correlation coefficients between the AUC of topology attribute parameters of the functional networks of the brain of patients with ESRD and the scores of the cognitive function of patients with ESRD. For patients with ESRD patients, the AUCs of γ and σ were positively correlated with the scores of the cognitive function (*p* < 0.0033, Bonferroni correction), while the AUC of C_p_, L_p_, E_global_, and E_local_ were not correlated with cognitive function scores (*p* > 0.0033, Bonferroni correction).

**TABLE 3:** Correlation analysis between AUC of topological parameters of the functional networks of the brain and scores of the cognitive function of patients with ESRD.

Parameter	γ	λ	σ	Cp	Lp	Eglobal	Elocal
*r*	0.607	0.166	0.531	0.194	0.139	−0.147	0.353
*P*	0.000	0.395	0.000	0.268	0.514	0.636	0.056

As shown in Tables 2, 3, the AUCs of γ and σ of patients with ESRD were significantly lower than those of normal controls, and they were positively correlated with the scores of the cognitive function. Therefore, we extracted the AUC of γ and σ as features. The extracted features were linearly fused (Wang et al., 2017; Jiao et al., 2019b; Li et al., 2020c), and then, GTA-SVRM, GTA-LSSVRM, GTA-WOA-SVRM, and GTA-WOA-LSSVRM separately performed regression prediction on the scores of the cognitive function of patients with ESRD.

The AUC of γ and σ with corresponding cognitive function scores of 45 patients with ESRD were used as a data set D by the hold-out method. It involves splitting D into two mutually exclusive sets. The AUCs of γ and σ with the scores of the cognitive function of 35 patients with ESRD admitted from February 2021 to July 2021 were used as the training set S. The AUCs of γ and σ with the scores of the cognitive function of 10 patients with ESRD admitted from July to September 2021 were used as test set T, that is, D = S∪T, S∩T = ∅. After the model is trained on S, the performance of the model is evaluated and measured on T. To evaluate the accuracy of the model, the root mean square error (RMSE), mean absolute error (MAE), and mean absolute percentage error (MAPE) were selected as the testing standards of the prediction accuracy. The smaller the RMSE, MAE, or MAPE, the higher the prediction accuracy of the model.

RMSE is defined as:


(16) $$\text{RMSE}=\sqrt{\frac{1}{n}\sum_{i=1}^{n}{\left({\hat{x}}_{i}-x_{i}\right)}^{2}}$$

MAE is defined as:


(17) $$\text{MAE}=\frac{1}{n}\sum_{i=1}^{n}\left|{\hat{x}}_{i}-x_{i}\right|$$

MAPE is defined as:


(18) $$\text{MAPE}=\frac{1}{n}\sum_{i=1}^{n}\left|\frac{{\hat{x}}_{i}-x_{i}}{x_{i}}\right|\times 100\%$$

where *n* is the number of predicted samples, $\hat{x}$ is the predicted scores of patients with ESRD in the test set, and *x* is the actual scores of patients with ESRD in the test set.

### Experimental Results

**FIGURE 3:**
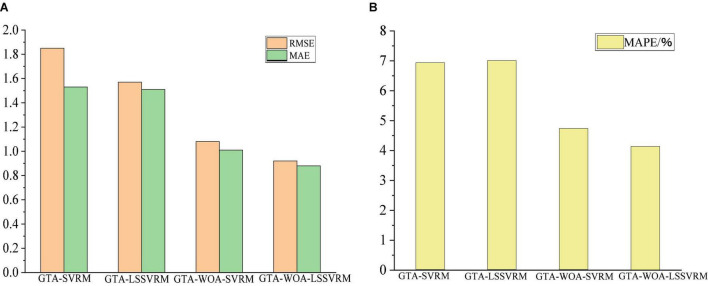
Prediction accuracies of various models. **(A)** RMSE and MAE **(B)** MAPE.

Table 4 shows the prediction accuracies of the various regression model for the scores of the cognitive function of patients with ESRD. As can be seen from the table, the prediction accuracy of GTA-WOA-LSSVRM is improved compared with those of GTA-SVRM, GTA-LSSVRM, and GTA-WOA-SVRM. The RMSE between the predicted scores of GTA-WOA-LSSVRM and the actual scores dropped to 0.92, which was 0.93, 0.65, and 0.16 points lower than those of GTA-SVRM, GTA-LSSVRM, and GTA-WOA-SVRM, respectively. The MAE between the predicted scores of GTA-WOA-LSSVRM and the actual scores is within 1, which was 0.65, 0.63, and 0.13 points lower than those of GTA-SVRM, GTA-LSSVRM, and GTA-WOA-SVRM, respectively. Compared with MAE, MAPE can further compare the relative errors of the model. The MAPE between the predicted scores of GTA-WOA-LSSVRM and the actual scores was 4.14%, which was 2.8, 2.87, and 0.6% lower than those of GTA-SVRM, GTA-LSSVRM, and GTA-WOA-SVRM, respectively. The bar chart in Figure 3 intuitively shows that the prediction accuracy of GTA-WOA-LSSVRM is better than those of GTA-SVRM, GTA-LSSVRM, and GTA-WOA-SVRM.

**TABLE 4:** Prediction accuracies of various models.

Predictive model	RMSE	MAE	MAPE%
GTA-SVRM	1.85	1.53	6.94
GTA-LSSVRM	1.57	1.51	7.01
GTA-WOA-SVRM	1.08	1.01	4.74
GTA-WOA-LSSVRM	0.92	0.88	4.14

**FIGURE 4:**
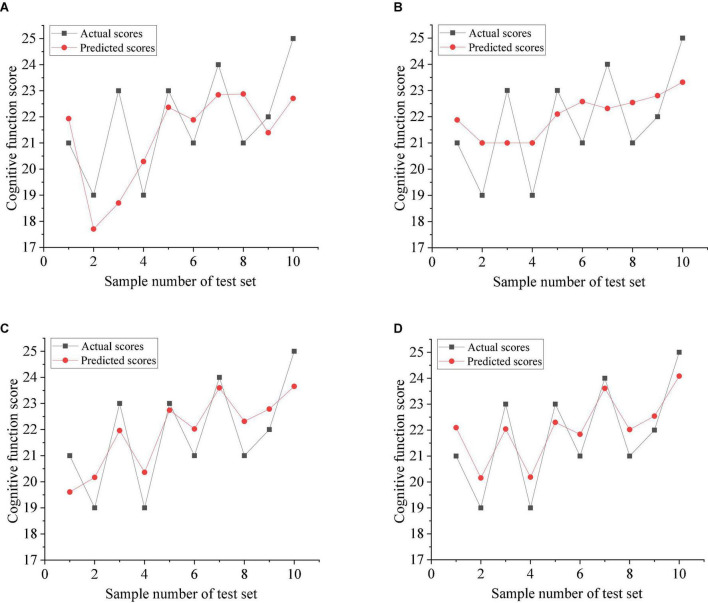
Actual scores and predicted scores of various models. **(A)** GTA-SVRM. **(B)** GTA-LSSVRM. **(C)** GTA-WOA-SVRM. **(D)** GTA-WOA-LSSVRM.

Figure 4 shows the comparison between the predicted scores of various models and the actual scores. The solid black line represents the actual scores, and the solid red line represents the predicted scores. As can be seen from the figure, GTA-WOA-SVRM and GTA-WOA-LSSVRM can fit well for most of the training samples, and the prediction results are closer to the real value, with high prediction accuracy. Moreover, the prediction results of 2–5 samples with relatively large score fluctuations are more accurate than those of GTA-SVRM and GTA-LSSVRM. It is worth noting that the strong fluctuation of scores results in a large error between the predicted results of GTA-SVRM and GTA-LSSVRM and the actual results, while the predicted results of GTA-WOA-SVRM and GTA-WOA-LSSVRM are relatively stable. This is due to the strong optimization ability of WOA, which optimizes penalty factors and kernel parameters in SVRM and LSSVRM and improves the generalization ability of the model.

### Discriminative Brain Regions

**FIGURE 5:**
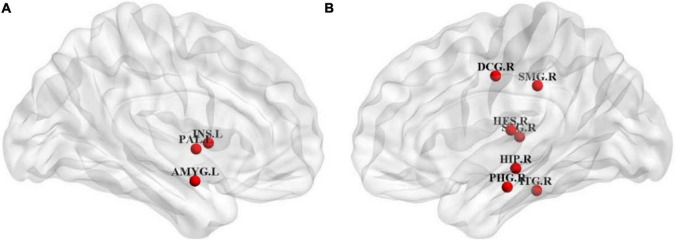
Distribution diagram of discriminative brain regions. **(A)** Coronal view of left hemisphere. **(B)** Coronal view of right hemisphere.

Node efficiency is mainly used to measure the information transmission capacity between one node and other nodes in the network (Li et al., 2020a; Ruby et al., 2020). To identify the key brain regions affecting the cognitive function, we calculated the *Pearson* correlation coefficients between the scores of the cognitive function in patients with ESRD and their node efficiency of 90 brain regions on the AAL template. Ten brain regions with the highest correlation with the scores of the cognitive function were selected as the discriminative brain regions. Table 5 shows their specific information. The BrainNet Viewer toolkit^1^ was used to visualize the discriminative brain regions and map them to the ICBM152 template, as shown in Figure 5.

**TABLE 5:** Discriminative brain regions.

Serial number	Brain regions	Abbreviations (L, left; R, right)
29	Left insula	INS.L
34	Right median cingulate and paracingulate gyri	DCG.R
38	Right hippocampus	HIP.R
40	Right parahippocampal gyrus	PHG.R
41	Left amygdala	AMYG.L
64	Right superior marginal gyrus	SMG.R
75	Left lenticular nucleus pallidum	PAL.L
80	Right heschl gyrus	HES.R
82	Right superior temporal gyrus	STG.R
90	Right inferior temporal gyrus	ITG.R

As shown in Table 5 and Figure 5, most of the selected brain regions have been widely considered to be possibly related to cognitive impairment. Among them, right hippocampus (HIP.R) and right parahippocampal gyrus (PHG.R) are related to the learning and memory function (Squire et al., 2007; Zhang et al., 2015). Right median cingulate and paracingulate gyri (DCG.R) is involved in cognitive control, and the structural damage to it may lead to abnormal cognitive behaviors (Shackman et al., 2011). Right inferior temporal gyrus (ITG.R) plays a role in hearing and is associated with memory and emotion, so its damage can cause personality changes (Bi et al., 2020b). Left insula (INS.L) and left amygdala (AMYG.L) are mainly involved in emotional processing, and they play important roles in the neuropathology of depression (Janak and Tye, 2015). In summary, the nodal efficiencies of these brain regions are strongly correlated with the scores of the cognitive function of patients with ESRD. It is basically consistent with the results of previous relevant studies (Jiao et al., 2020a, 2021a; Peng et al., 2020).

## Discussion

In this article, we have proposed a novel GWLS model called GTA-WOA-LSSVRM to optimally predict the scores of the cognitive function of patients with ESRD. Comparatively, although the prediction accuracy is improved limitedly, the operation efficiency of GTA-LSSVRM is higher than that of GTA-SVRM. As an improvement on SVRM, LSSVRM changes the inequality constraint in the SVRM model into equality constraint and transforms the solution of the quadratic programming problem into the solution of linear equations, so the calculation is faster. The prediction accuracy of GTA-WOA-SVRM and GTA-WOA-LSSVRM is significantly higher than those of GTA-SVRM and GTA-LSSVRM. It is due to the strong optimization ability of WOA, which optimizes the strategy of selecting kernel function parameters in SVRM and LSSVRM, thus improving the generalization ability of the model and helping to fit the values with large fluctuations effectively. In the clinical diagnosis, it is often necessary to predict the scores of the cognitive function in large number of patients with ESRD for research, and the scores of different patients vary greatly. Therefore, GTA-WOA-LSSVRM has taken both work efficiency and accuracy into account.

During extracting features, we found that the AUCs of γ and σ in patients with ESRD were significantly lower than those in normal controls. γ is an important indicator to measure the connection tightness between nodes of the functional networks of the brain. It mainly reflects the local information processing and transmission ability of networks. Accordingly, γ is related to the short-range connections between adjacent brain regions, and these brain regions can mediate modular information processing (Jiao et al., 2019a, 2020b). In patients with ESRD, the reduction of γ means the modular information processing capacity of the functional network of the brain is reduced, which leads to the impairment of the local information processing and transmission capacity of the network. σ is mainly used to measure the small-world attribute of the functional networks of the brain (Bassett and Bullmore, 2017). The characteristics of the optimized network topology of patients with ESRD are obviously weakened than those of normal controls. Different from γ and σ, E_global_, λ, and L_p_ mainly reflect the information transmission and integration ability of the functional networks of the brain at the global level in patients with ESRD. This indicates that patients with ESRD only show impaired local network information processing and transmission capacity (i.e., functional separation), while the global level of the long-range connectivity and information transmission capacity (i.e., functional integration) is not significantly impaired. This phenomenon may also have something to do with the compensation mechanism of the network.

Based on this, it has been suggested that the functional networks of the brain of patients with ESRD may maintain their global information transmission ability through the remodeling mechanism before clinically visible cognitive impairment, thus preventing a sharp decline in the cognitive function (Wei et al., 2018; Cheng et al., 2019). This provides a new perspective and potential imaging biomarkers for understanding the underlying pathophysiological mechanisms of cognitive impairment in patients with ESRD.

However, there are still some deficiencies in our study. First, the influence of dialysis methods (such as hemodialysis and peritoneal dialysis) on the functional networks of the brain of patients with ESRD was not evaluated (Li et al., 2021b). Second, more common methods are applied to the feature extraction and feature selection. In the following work, we will try to improve the existing feature extraction and feature selection methods, so that our new model can better mine the information of functional networks of the brain, enhance the prediction ability of the model, and assist doctors in diagnosis more effectively. In addition, the number of experimental samples in this study is limited. Although the evaluation performance of the model can be reflected to some extent, more extensive data will be more convincing. In future experiments, it is necessary to collect more fMRI, DTI, DKI, and other multimodal data and fuse the data in different modes to build brain networks with structural connections and functional connections (Wang et al., 2019; Bi et al., 2020a, 2021). Finally, the topology attributes of fused networks will be selected to improve the accuracy on predicting the scores of the cognitive function of patients with ESRD.

## Data Availability Statement

The original contributions presented in the study are included in the article/supplementary material, further inquiries can be directed to the corresponding author/s.

## Ethics Statement

The studies involving human participants were reviewed and approved by the Medical Ethics Committee of Changzhou Second People’s Hospital. The patients/participants provided their written informed consent to participate in this study. Written informed consent was obtained from the individual(s) for the publication of any potentially identifiable images or data included in this article.

## Author Contributions

YZ: formal analysis, methodology, and writing—original draft. ZX: software and visualization. JZ: data curation. HS: conceptualization, methodology, and writing—review and editing. ZJ: methodology, supervision, and writing—review and editing. All authors contributed to the article and approved the submitted version.

## Conflict of Interest

The authors declare that the research was conducted in the absence of any commercial or financial relationships that could be construed as a potential conflict of interest.

## Publisher’s Note

All claims expressed in this article are solely those of the authors and do not necessarily represent those of their affiliated organizations, or those of the publisher, the editors and the reviewers. Any product that may be evaluated in this article, or claim that may be made by its manufacturer, is not guaranteed or endorsed by the publisher.

## Footnotes

1
https://www.nitrc.org/projects/bnv
